# Clinical efficacy analysis of intelligent pressure-controlled ureteroscopy combined with thulium laser in the treatment of isolated upper urinary tract urothelial carcinoma

**DOI:** 10.3389/fonc.2024.1406031

**Published:** 2024-10-15

**Authors:** Hua Chen, Jiansheng Xiao, Jiaqi Ge, Tairong Liu

**Affiliations:** Department of Urology, Ganzhou People’s Hospital, Jiangxi Medical College, Nanchang University, Ganzhou, Jiangxi, China

**Keywords:** intelligent pressure-controlled, thulium laser, upper tract urothelial carcinoma, ureteroscopy, treatment

## Abstract

**Objective:**

This study aims to investigate the clinical treatment effect of intelligent pressure-controlled ureteroscopy combined with thulium laser for patients with isolated kidney upper tract urothelial carcinoma (UTUC).

**Methods:**

This study employed a retrospective analysis approach and focused on six patients with isolated kidney UTUC admitted to our hospital from June 2018 to May 2023, who underwent tumor resection surgery using intelligent pressure-controlled ureteroscopy combined with thulium laser. We collected the perioperative clinical data of these six patients and conducted statistical analysis of the treatment effects.

**Results:**

The surgeries of all six patients were completed smoothly, without incidents of surgery termination due to significant bleeding. Postoperative pathology revealed that four patients had low-grade non-invasive papillary urothelial carcinoma, while the other two patients had high-grade invasive urothelial carcinoma. During follow-up period, one patient had a renal pelvis recurrence three months after the surgery, and subsequently underwent thulium laser resection. Additionally, another patient experienced bladder recurrence eight months after the surgery and received transurethral resection of bladder tumor (TURBT) for treatment. The remaining four patients did not experience tumor recurrence during the follow-up.

**Conclusion:**

For patients with isolated kidney associated with UTUC, intelligent pressure-controlled ureteroscopy combined with thulium laser represents a feasible treatment option, with good therapeutic effects for low-risk upper tract urothelial carcinoma.

## 1 Introduction

Globally, upper tract urothelial carcinoma (UTUC) is a relatively rare tumor in clinical practice, with an annual incidence rate of approximately 2 cases per 100,000 people in Western countries (1). However, in China, due to specific geographical factors and widespread use of aristolochic acid-containing drugs, the incidence of UTUC is significantly higher than in European countries (2). In treatment of UTUC, radical nephroureterectomy is considered the gold standard treatment for this disease (3). However, performing radical nephroureterectomy (RNU) on patients with solitary kidney and UTUC results in loss of renal function, leading to lifelong dialysis dependency. This significantly impacts long-term survival rates and quality of life. Common complications after RNU include infection, bleeding, thrombosis, and chronic kidney disease due to loss of renal function. These complications occur more frequently in patients with a solitary kidney, as their remaining renal function cannot compensate, failing to significantly improve their overall survival rate (4, 5).

According to the European Association of Urology guidelines on UTUC, conservative treatment is the preferred strategy for patients with a solitary kidney. This approach aims to preserve as much renal function as possible while effectively controlling the tumor. Conservative treatments include local instillation using drugs such as radiotherapy and chemotherapy to reduce the risk of recurrence, and local tumor resection using ureteroscopes or flexible scopes combined with lasers (1, 6, 7). However, local tumor resection using ureteroscopes or flexible scopes combined with lasers requires early repeated ureteroscopic examinations. Studies have shown that early repeated ureteroscopic examinations can effectively detect tumor recurrence and help assess the aggressiveness of the disease, which is crucial for risk stratification in patients undergoing endoscopic treatment for UTUC (8). Therefore, with the informed consent of the patients, we employed intelligent pressure-controlled transurethral flexible ureteroscopy combined with thulium laser for surgical resection in patients with solitary kidney and UTUC, followed by early regular follow-up ureteroscopic examinations. This approach has yielded certain clinical benefits.

## 2 Materials and methods

### 2.1 Study population

This study was approved by the Ethics Committee of Ganzhou People’s Hospital, and informed consent was obtained from all patients, who each signed a consent form. We conducted a retrospective analysis on six cases of isolated kidney UTUC patients admitted to our hospital from June 2018 to May 2023.All patients underwent tumor resection using intelligent pressure-controlled ureteroscopy combined with thulium laser, and perioperative clinical data were collected for effectiveness assessment and analysis. This study involved six patients, with four males and two females. There were four cases of renal pelvis tumors and two cases of upper ureteral tumors, all with tumor diameters less than 3 cm. The average preoperative creatinine level of the patients was 98.2 umol/L (range 65-135 umol/L), and the average age of the 6 patients was 58.6 years (ranging from 37 to 76 years). Three patients had hypertension, one had diabetes, and two had coronary heart disease. Four patients were admitted for painless gross hematuria, while the remaining two were diagnosed during routine physical examinations. Preoperative enhanced CT scans showed that all six patients had solitary tumors with diameters less than 3cm.Urine cytology examination revealed urothelial carcinoma cells in two patients. Inclusion criteria for this study: 1. Patients diagnosed with renal pelvis lesions suggestive of UTUC by imaging examinations such as CT or MRI; 2. Patients with absent contralateral kidney or a contralateral kidney GFR <5, indicating non-functional kidney; 3. Patients without distortion or stenosis of the affected ureter, allowing for intelligent pressure-controlled flexible ureteroscopy surgery; 4. Lesions in the affected renal pelvis or calyces categorized as high-risk according to the Chinese Urological Association guidelines. Exclusion criteria: 1. Patients with tumors <2 cm without associated hydronephrosis, categorized as low-risk UTUC; 2. Patients with advanced-stage tumors infiltrating the renal parenchyma or renal fat, making complete resection via flexible ureteroscopy unlikely; 3. Patients with normal or relatively good function of the contralateral kidney; 4. Patients with poor physical condition or bleeding disorders unable to tolerate surgery.

### 2.2 Surgical methods

**Figure 1:**
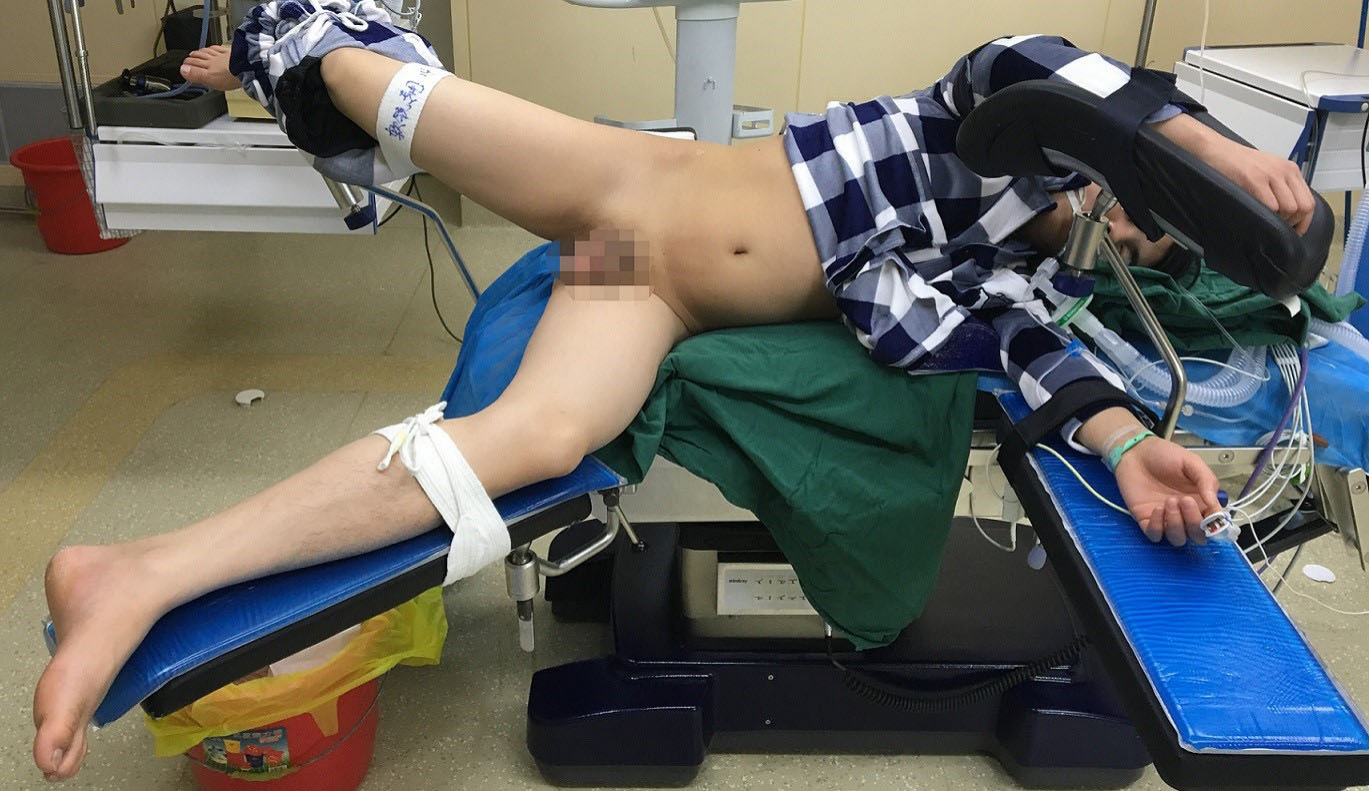
The healthy side lying on a running position.

**Figure 2:**
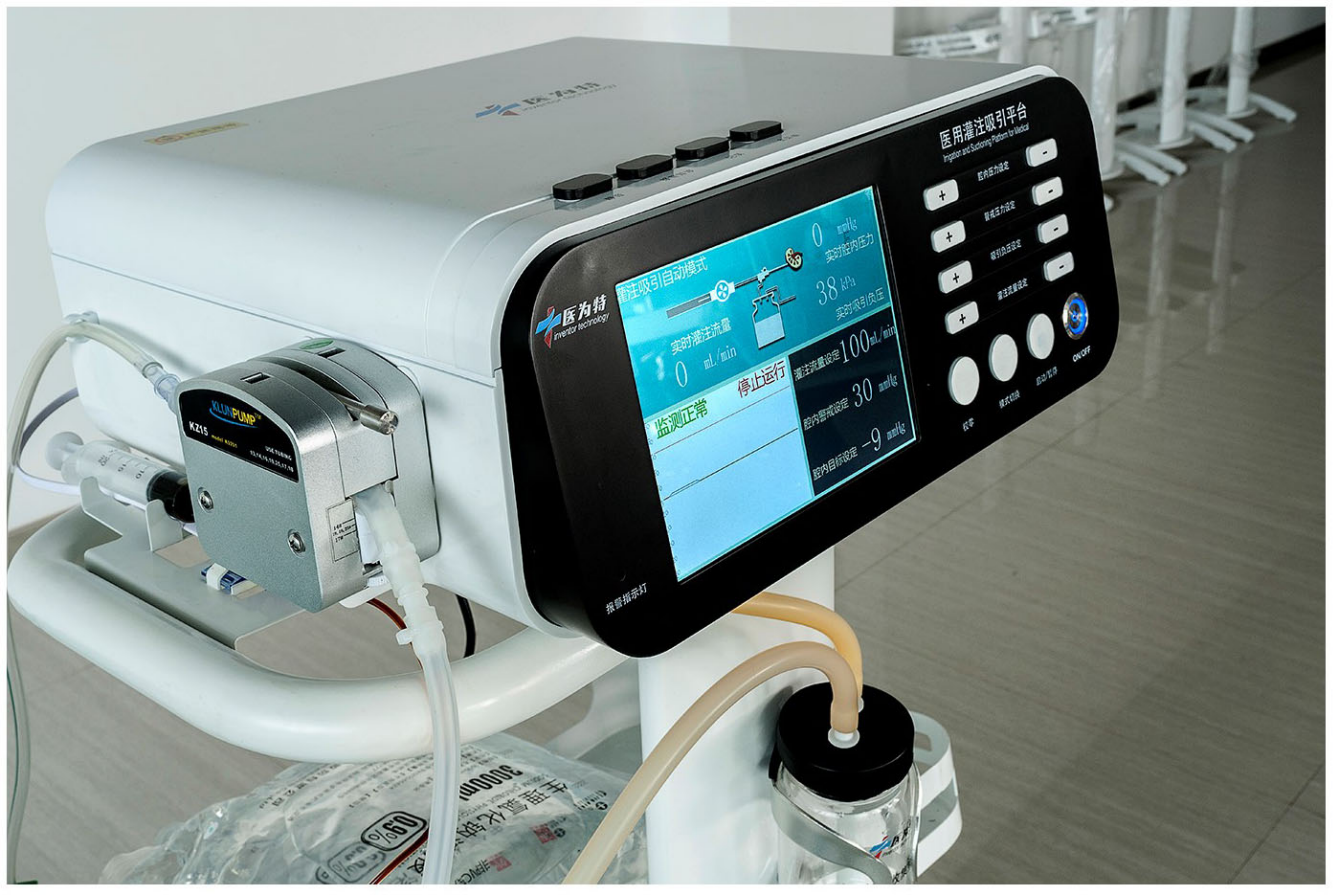
Medical perfusion attraction platform.

**Figure 3:**
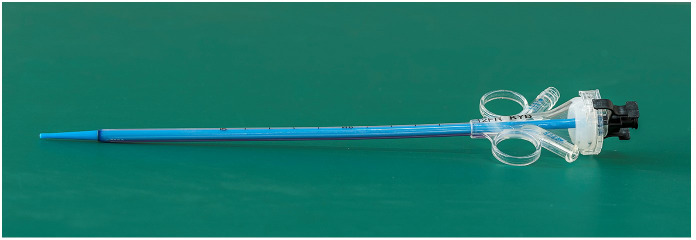
Disposable sterile ureteral catheter introducer sheath.

**Figure 4:**
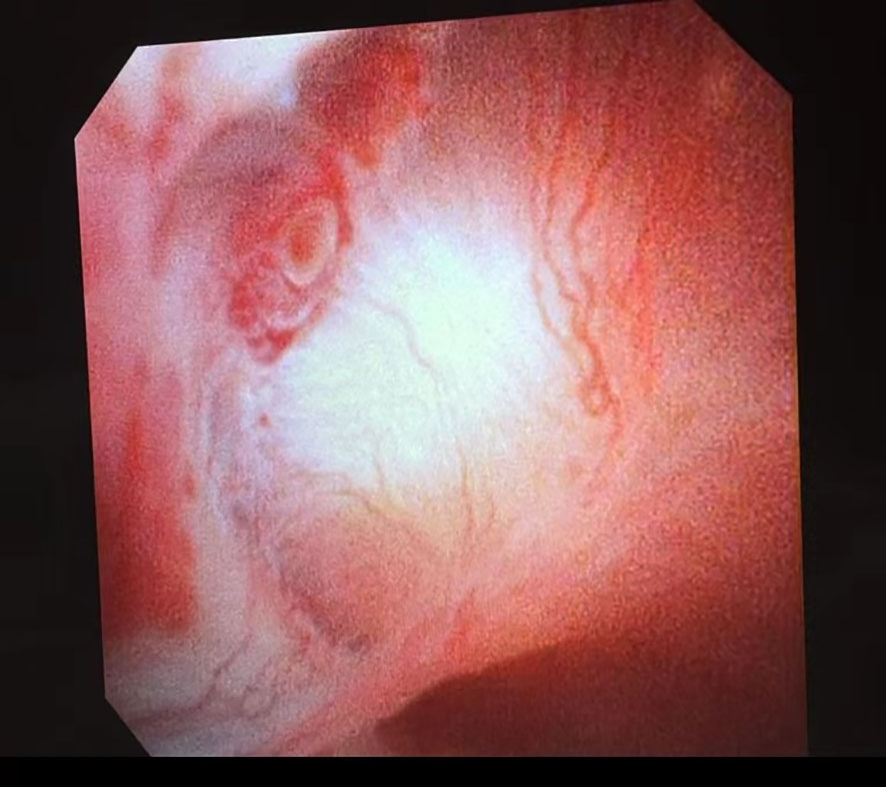
Tumor at the top of the renal pelvis.

**Figure 5:**
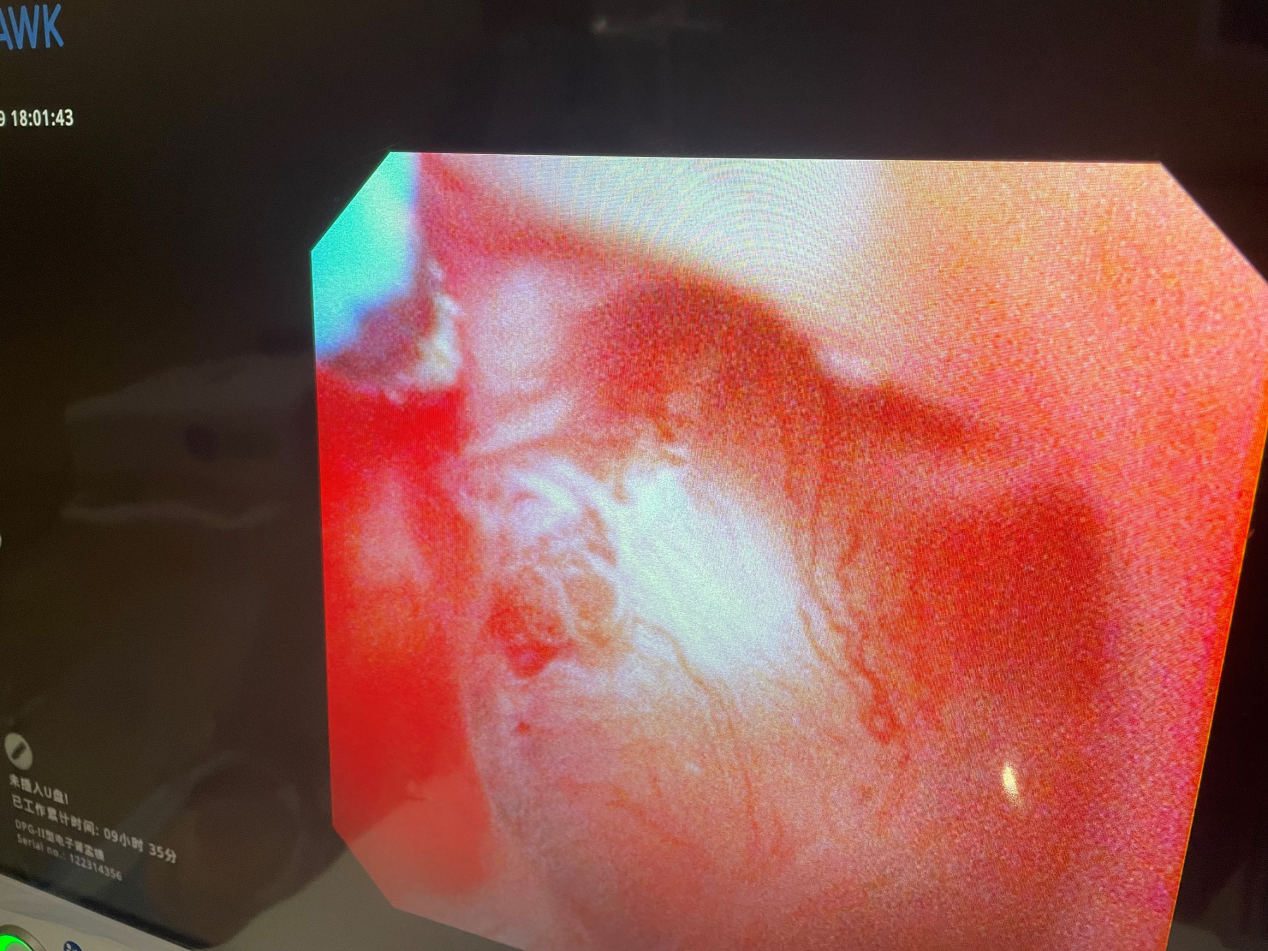
Thulium laser resection of the tumor.

**Figure 6:**
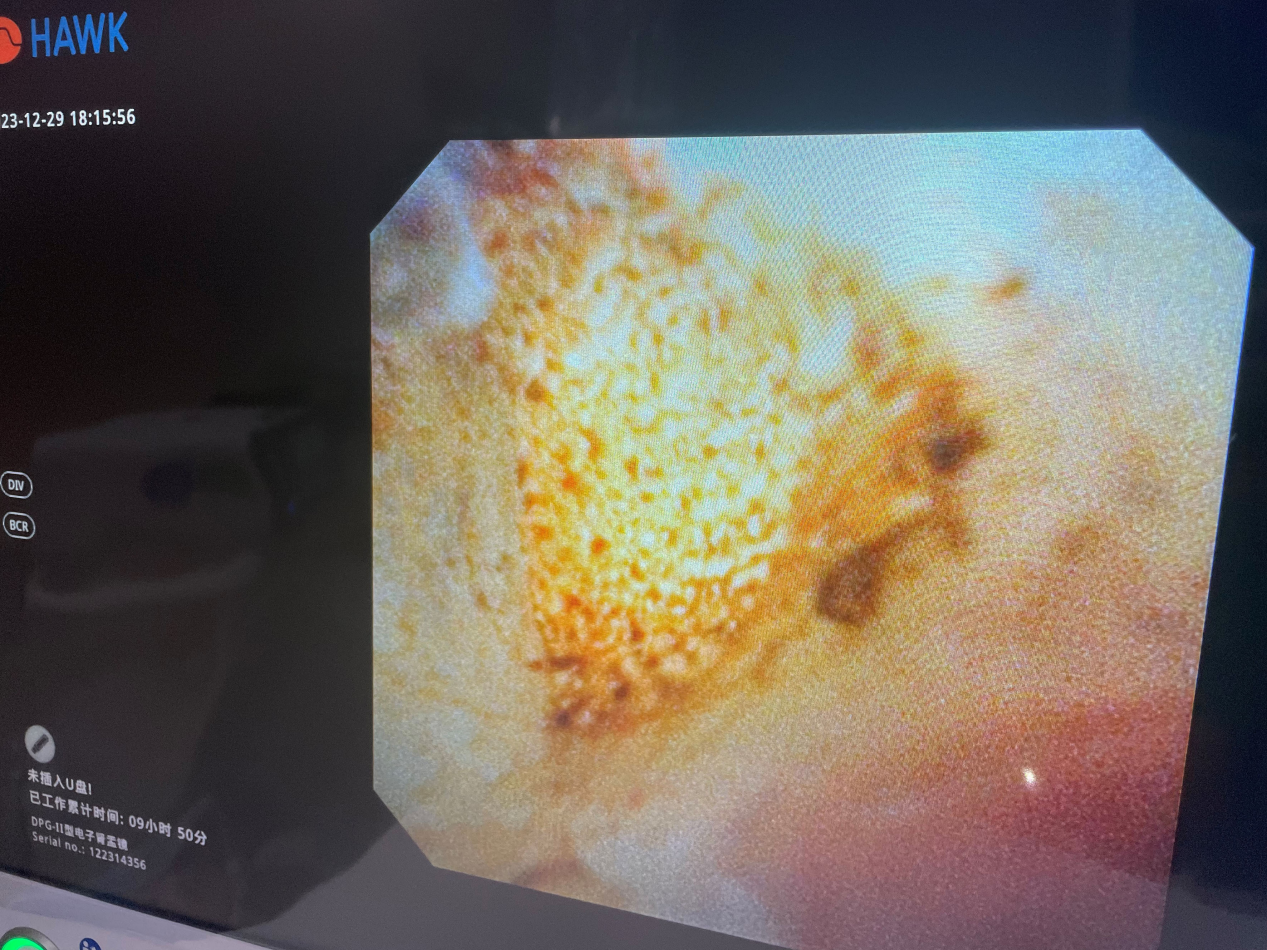
The tumor has been removed.

All patients underwent surgery under general anesthesia in a healthy-side oblique supine position (**Figure 1**). First, a ureteroscope (7-8.5 Fr, Wolf, USA) was introduced into the patient’s ureter and renal pelvis under low-pressure, low-flow perfusion. After confirming the ureter was patent without significant stenosis or distortion, a zebra guidewire was inserted. Along the guidewire, a 12-14 Fr integrated pressure-measuring suction ureteroscope sheath was introduced (**Figure 2**). The sheath’s pressure measurement and suction ports were connected to the intelligent perfusion suction pressure control platform (**Figure 3**) using pressure and suction tubes, respectively. The platform zeroed the intracavitary pressure and set the intraoperative intracavitary pressure control value (-10 mmHg) and perfusion flow rate (50 ml/min). Then, an electronic flexible ureteroscope (11278VU 8.5 Fr, Storz, Germany) was used. The ureteroscope was connected to the platform perfusion pump, and the platform’s automatic switch was activated. The platform could automatically adjust the negative pressure suction capability based on the set intracavitary pressure control value. The ureteroscope was introduced into the sheath, and after locating the tumor (**Figure 4**), a 200 μm thulium laser (Rekon, China) with a power setting of 25 W was used to enucleate the tumor from the periphery to the center (**Figure 5**). The excised tumor tissue was extracted intact into a specimen collection bottle through the withdrawal and suction method. The ureteroscope was reintroduced to treat the wound. If any obvious tumor tissue was found around the wound, it was ablated and vaporized using the thulium laser, and thorough hemostasis was ensured. After confirming no significant tumor tissue or bleeding (**Figure 6**), the flexible ureteroscope and ureteral sheath were withdrawn, a 6 Fr double-J stent was placed, and a urinary catheter was inserted.

### 2.3 Postoperative management and follow-up

The double-J stents were left in place for 2 weeks in all 6 patients. All patients received 30 mg of pirarubicin bladder instillation within 24 hours postoperatively, with a duration of 30 minutes. Two patients with high-grade infiltrating urothelial carcinoma received “gemcitabine plus cisplatin” chemotherapy as adjuvant therapy, while four patients with low-grade non-invasive papillary urothelial carcinoma did not undergo adjuvant chemotherapy. Postoperatively, all six patients were followed up, with a follow-up rate of 100%. The follow-up period ranged from 6 to 39 months, with an average of (16.57 ± 4.69) months. During follow-up period, patients presenting with symptoms such as hematuria should immediately return to the hospital for enhanced CT of the urogenital system and cystoscopy. If tumor recurrence is detected, further treatment should be initiated promptly. Subsequent arrangements include returning to the hospital every three months for enhanced CT of the urogenital system, complete blood count, renal function tests, urine analysis, and cystoscopy for routine examinations.

## 3 Results

The surgeries of all 6 patients were successfully completed, with an average operation time of (43 ± 13.45) minutes, and an estimated average intraoperative blood loss of (10 ± 2.26) milliliters. Postoperative pathological examination results showed that 4 patients had low-grade non-invasive papillary urothelial carcinoma, and 2 patients had high-grade invasive urothelial carcinoma. During the follow-up period, one patient with high-grade invasive urothelial carcinoma was found to have renal pelvis recurrence 2 months after the surgery and underwent thulium laser resection treatment. Additionally, one patient with low-grade non-invasive papillary urothelial carcinoma was found to have bladder recurrence 8 months after the surgery, and after transurethral resection and intravesical chemotherapy, the condition was well controlled. The preoperative average serum creatinine level was 98.2umol/L (range 65-135umol/L), while it was 102.4umol/L (range 68-168umol/L) postoperatively. There was no significant statistical difference in serum creatinine changes pre- and postoperatively (p<0.05). The recurrence rate of low-grade non-invasive urothelial carcinoma was 14%, with a relatively good prognosis, while the postoperative recurrence rate for patients with high-grade invasive urothelial carcinoma was as high as 50%.

## 4 Discussion

In recent years, due to unique geographical factors and the use of aristolochic acid medications, the incidence of UTUC in China accounts for approximately 18% of urothelial carcinoma cases, higher than the 5%-10% in Western countries (9). Radical nephroureterectomy is considered the “gold standard” for treating this disease, but for some patients with a solitary kidney or impaired renal function, undergoing radical surgery would lead to lifelong hemodialysis, severely impacting the quality of life (10). Therefore, for such patients, minimally invasive tumor resection via a natural orifice approach is considered an alternative treatment modality (11). With the advancement of ureteroscopy and laser technology, minimally invasive surgery through natural orifice for treating UTUC is becoming increasingly common (12–14).

During the procedure, the intelligent pressure-controlled ureteroscope utilized a self-developed intelligent pressure control device, which includes an intelligent irrigation and suction pressure monitoring platform and an integrated pressure-sensing ureteroscope insertion sheath for guidance. The functions of the intelligent pressure control platform include: setting the required irrigation flow rate for surgery, controlling the renal pelvic pressure, setting the warning pressure for renal pelvic pressure, and the upper limit of renal pelvic pressure. It also receives pressure data from the ureteroscope suction sheath for renal pelvic pressure monitoring and, utilizing pressure feedback control technology based on the set renal pelvic pressure control value, automatically adjusts negative pressure suction to maintain the renal pelvic pressure within the predetermined safe range (15, 16). Internal diameter of the ureteroscope insertion sheath is 12Fr, the external diameter is 14Fr, and the length ranges from 20 to 45cm. It integrates pressure sensing and suction devices, enabling connection to the pressure control instrument for real-time monitoring of renal pelvic pressure during stone retrieval using negative pressure suction. The sheath is made of transparent material, facilitating observation of the surroundings during surgery and aiding in tumor localization.

Previous studies have shown that among UTUC patients who underwent minimally invasive treatment via the urethra, the postoperative upper urinary tract tumor recurrence rate was 65% (range 15% to 90%), and the bladder tumor recurrence rate was 44% (range 19% to 70%). In comparison, patients undergoing standard radical surgery had a bladder recurrence rate of 11% to 36% (17, 18). Regarding survival rates, studies have indicated that patients undergoing nephron-sparing surgery had a 2-year overall survival rate of 35% to 100% and a tumor-specific survival rate of 70% to 100% (19, 20). In this study, we utilized a self-developed intelligent pressure control device in conjunction with ureteroscopy and thulium laser therapy for six elderly UTUC patients. Postoperative pathological results revealed that 4 cases were low-grade non-invasive papillary urothelial carcinoma, of which 1 case had a bladder tumor recurrence 18 months after the surgery. Additionally, 2 cases were high-grade invasive urothelial carcinoma, with 1 case experiencing renal pelvis recurrence 3 months postoperatively. During the follow-up period, none of the six patients experienced distant organ metastasis, and they were still in good health with a tumor-specific survival rate of 100%.

In this study, we achieved high-volume irrigation under ureteroscopy using an intelligent pressure control device, which continuously applied negative pressure to actively remove irrigating fluid containing blood and tumor cells in real-time, promoting fluid circulation within the cavity. This approach not only maintained a clear surgical field but also prevented iatrogenic tumor dissemination due to leakage of irrigating fluid caused by high renal pelvis pressure, and reduced laser-related collateral damage resulting from severe hematuria. Thulium laser is currently the shallowest penetrating laser in medical use, with a depth of penetration of only 0.2 mm. It enables precise tissue cutting, rapid hemostasis, and efficient vaporization (21). As a continuous-wave laser, compared to conventional holmium lasers, thulium lasers have a faster tissue heating rate, better vaporization effect, and superior hemostatic performance (22). When performing thulium laser ablation and vaporization therapy, we used a low power setting of 25W.arefully cutting along the tumor edge towards the tumor base and laser cauterizing visible vessels under the scope to prevent bleeding, thereby occluding the blood supply around the tumor and avoiding severe bleeding caused by direct tumor excision, which may impact surgical outcomes. When resecting upper ureteral tumors, avoid cutting too deeply to prevent ureteral perforation that could facilitate tumor implantation and metastasis, excessive damage to the muscle layer of the ureter may lead to ureteral stricture. In this study, leveraging the advantages of two devices and unique positioning, none of the 6 patients experienced major bleeding, ureteral perforation, tearing, renal rupture, or severe infection during the surgical procedures. During the follow-up period, no distant organ metastases to the liver, lungs, or other organs were observed in these 6 patients. Therefore, it can be concluded that combining intelligent pressure-controlled ureteroscopy with thulium laser therapy for upper urinary tract urothelial carcinoma in solitary kidney patients is a safe and reliable treatment method. However, following UTUC nephron-sparing treatment, patients require long-term intensive imaging and endoscopic follow-up (23). Hence, the overall treatment costs and duration are also important considerations.

This study has the following limitations: Firstly, the number of cases is relatively small, the follow-up period is short, and it is a retrospective study. Although the results show some efficacy, the small sample size introduces selection bias, which is an unavoidable limitation of retrospective studies. Additionally, the equipment used is currently only available in China and has not yet been approved by the FDA, potentially leading to regional limitations of the findings. Furthermore, during intraoperative procedures, the completeness of tumor ablation was judged solely based on endoscopic appearance, which cannot accurately determine whether the tumor was entirely resected, thus affecting the assessment of postoperative tumor staging. Lastly, intraluminal nephron-sparing surgery for solitary kidney high-risk urothelial carcinoma is still in the exploratory stage, with large-scale clinical studies being rare both domestically and internationally. Despite these limitations, this small retrospective study suggests that this technique can achieve clinical efficacy in managing relatively early high-risk upper tract urothelial carcinoma. We plan to apply for a multi-center clinical study in the future to further demonstrate the safety and efficacy of this technique.

In summary, for patients with UTUC, intelligent pressure-controlled flexible ureteroscopy combined with thulium laser is an effective treatment option that clinicians can consider. However, close imaging and endoscopic follow-up are necessary after the procedure.

## Data Availability

The original contributions presented in the study are included in the article/supplementary material. Further inquiries can be directed to the corresponding author.
